# Fibrinogen depletion prevents non-dissociable cell aggregates of feeder-derived NK cell products

**DOI:** 10.3389/fimmu.2026.1887148

**Published:** 2026-09-16

**Authors:** Jiaoqi Wu, Jianhua Chen, Mingzhu Victoria Huang, Caihua Huang, Wenyi Lee, Jian Wang, Qi Liu, Shuo Tian, Wenlin Huang

**Affiliations:** 1 Guangzhou Doublle Bioproduct Co., Ltd, Guangzhou, China; 2 Guangdong Provincial Engineering Laboratory of Novel Tumor Vaccine, Guangzhou, China; 3 Department of Molecular Cell and Developmental Biology, University of California, Los Angeles, Los Angeles, CA, United States

**Keywords:** cell aggregation, feeder cells, fibrinogen, natural killer cells, serum substitute

## Abstract

**Introduction:**

Natural killer cells, as critical components of the innate immune system, have shown a promising potential in cancer immunotherapy. Currently, irradiated genetically modified feeder cells are widely used to produce large quantities of highly cytotoxic NK cells. However, it is frequently overlooked that NK cell products prepared in this way tend to form non-dissociable and macroscopic aggregates during storage and transportation, posing a significant challenge in clinical applications. The formation of cell aggregates not only compromises cell yield and quality but also raises concerns for vascular occlusion or unexpected immune-related toxicity upon infusion.

**Methods:**

To address this issue, we systematically investigated key factors contributing to aggregate formation throughout the NK cell manufacturing process. Primary NK cells isolated from cord blood or peripheral blood were expanded using feeder cells. We evaluated the impact of harvesting parameters (centrifugal force, final cell concentration, filtration pore size, and human serum albumin concentration), anticoagulant supplementation, feeder cells, and plasma fibrinogen content on aggregate formation. Aggregate formation was monitored and quantified microscopically post-formulation. Meanwhile, NK cell quality attributes, including cell apoptosis, purity, proliferation and cytotoxic function, were assessed by cell counter and flow cytometry to confirm the quality of NK cell products.

**Results:**

Our findings suggest that the coexistence of feeder cells and fibrinogen in the culture medium jointly induces aggregation in the final NK cell product. Replacing autologous plasma with a fibrinogen-free serum substitute can effectively prevent the aggregation, without impairing NK cell expansion fold, phenotypic stability, viability, or cytotoxic potency.

**Conclusions:**

In summary, our study unveils the phenomenon and mechanism of cell aggregation in feeder-derived NK cell products, and establishes an optimized protocol for NK cell manufacturing, which supports the clinical feasibility of feeder cell-derived NK cell therapies.

## 1 Introduction

Natural killer (NK) cells are essential components of the innate immune system and play a pivotal role in immune surveillance against malignancies (1–3). In contrast to adaptive lymphocytes, NK cells can directly recognize and eliminate transformed or virus-infected cells without the requirement of prior sensitization (4). The ability to mount rapid, non-antigen-specific cytotoxic responses makes them particularly attractive candidates for off-the-shelf cancer immunotherapy (5). However, the translation of NK cell-based therapies into widespread clinical application remains constrained by the difficulty of generating sufficient numbers of highly functional NK cells under current good manufacturing practice (cGMP) conditions (6).

A variety of expansion strategies have been explored to overcome this limitation, including multiple cytokine cocktails (7) and membrane-bound cytokine-modified feeder cells (8, 9). Among these, feeder cell-based platforms have emerged as the most widely adopted approach, due to their robust capacity to support extensive NK cell proliferation (4, 10). The most commonly employed feeder line, K562, is genetically modified to stably express membrane-bound stimulatory molecules such as IL-21 and IL-15, which provide sustained activation signals (11–14). Prior to co-culture, feeder cells are rendered replication-incompetent via irradiation, thereby ensuring selective outgrowth of NK cells under the synergistic influence of multiple activating signals (15, 16). This strategy has enabled the generation of large numbers of NK cells suitable for both early-phase clinical studies and commercial large-scale production (17, 18).

In our own efforts, we successfully established a feeder-based expansion system for primary NK cells, achieving a total cell expansion of approximately 500-fold and an NK-specific expansion of approximately 10 000-fold, with the expanded NK cells displaying robust tumor-cytolytic activity. However, unexpected macroscopic and non-dissociable cell aggregates were consistently observed in all the final NK cell products within hours, irrespective of donor source, feeder line, or expansion duration. These aggregates increased progressively in size and number during standard 2–8 °C storage and could not be dispersed by gentle shaking. To our knowledge, this phenomenon has not been previously documented in the context of feeder-expanded NK cells, as previous studies mainly focus on the cell yield, purity and cytotoxicity, with little attention of cell stability during storage or transportation. Nevertheless, its consistent emergence and potential implications for product quality, functional consistency, and infusion safety warrant urgent attention.

Accordingly, this study aimed to unveil the cause of cell aggregates formation in feeder-expanded NK cell products, which would be beneficial to optimize the culture parameters and medium formulations. And these insights would provide a theoretical and technical basis for the development of robust and scalable NK cell manufacturing platforms, and thus improve therapeutic efficacy and safety of NK cell-based adoptive immunotherapy.

## 2 Materials and methods

### 2.1 Feeder cells construction

K562 cells (National Collection of Authenticated Cell Cultures, China) engineered to express 4-1BBL and mbIL-21 were generated via lentiviral transduction and subsequently sorted using a CytoFLEX SRT cell sorter. K562-4-1BBL-mbIL-21 cells were cultured in RPMI 1640 medium (Gibco, USA) supplemented with 10% heat-inactivated fetal bovine serum (FBS; Gibco, USA) at 37 °C and 5% CO_2_. Expression of 4-1BBL and mbIL-21 was confirmed by flow cytometry using 4-1BBL (BioLegend, USA) and IL-21 (BioLegend, USA) antibodies. Before application, K562 cells were irradiated with a dose of 100Gy using a X-Ray irradiator.

### 2.2 NK cell expansion

Mononuclear cells (MNCs) and autologous plasma were isolated from umbilical cord or peripheral blood of healthy donors (Milecell, China). All NK-cell-based biological replicates were performed using cells obtained from distinct human donors. Plasma was inactivated at 56 °C for 30 min and subjected to one freeze-thaw cycle. Subsequently, the supernatant was collected by centrifugation at 2500 rpm for 10 min. Isolated mononuclear cells and irradiated K562-4-1BBL-mbIL-21 feeder cells were co-cultured at a 2:1 ratio in lymphocyte serum-free medium KBM581 (Corning, USA) supplemented with 5% heat-inactivated autologous plasma or fibrinogen-depleted cell culture supplement (Helios Bioscience, USA), and 200 IU/mL IL-2 (PeproTech, USA). On day 3 of culture, all cells were collected, centrifuged at 300×g for 5 min, and resuspended in fresh medium of equal volume. Fresh medium was replenished daily to maintain a cell density between 1.0 and 1.5 × 10^6^ cells/mL. On day 7, a second stimulation was performed by adding irradiated K562-4-1BBL-mbIL-21 cells at NK-to-feeder ratios of 10:1, 20:1, or 40:1. Subsequently, the concentration of plasma or cell culture supplement in the medium was reduced from 5% to 1%, and cell density was maintained between 1.0 and 1.5 × 10^6^ cells/mL. The cells were harvested on day 13, and the expansion fold was calculated based on the absolute number of NK cells on day 13 relative to day 0. For comparison, two additional commercially available feeder cells (Nuwacell Biotech, China; Yipin Biotech, China) were used according to the manufacturers’ instructions. Cytokine-expanded NK cells were generated using an umbilical umbilical cord blood NK cell induction kit and Alys505NK-AC/EX medium (BaSO Cell, China) strictly following the manufacturer’s protocol, with fresh medium supplemented every 2–3 days to maintain cell density between 1.0 and 1.5 × 10^6^ cells/mL. The cells were collected on 15–20 days. Expansion fold was calculated as:


Expansion fold=(Final cell number)/(Initial cell number)

### 2.3 NK cell harvest

NK cells were harvested by centrifugation at 300–800×g for 10 min and washed twice with normal saline. The washed cells were resuspended in normal saline containing 1.5% HSA (Takeda, Japan) at a final density of 2–4 × 10^7^ cells/mL and filtered through a 40–100 μm cell strainer (Corning, USA). The resulting cell suspension was transferred to a 150 mL cell culture bag and stored at 4 °C, with regular monitoring at indicated time points.

### 2.4 Flow cytometry analysis

Apoptosis was examined using the Annexin V-FITC/PI Apoptosis Detection Kit (Keygen Biotech, KGA1102, China) according to the manufacturer’s instructions. NK cells were stained with Annexin V-FITC and PI working solution at 4 °C in the dark for 10 min. Early apoptotic (Annexin V^+^/PI^−^) and late apoptotic/necrotic (Annexin V^+^/PI^+^) populations were quantified, and the total apoptosis rate was calculated as the sum of these two populations.

For surface marker detection, cells were stained with fluorophore-conjugated antibodies targeting CD3 (BioLegend, clone OKT3, USA), CD56 (BioLegend, clone W22097A, USA), NKG2D (BioLegend, clone 1DD11, USA), NKp44 (BioLegend, clone P44-8, USA), NKp46 (BioLegend, clone 9E2, USA) and CD16 (BioLegend, clone 3G8, USA) at 4 °C for 20–30 min. For intracellular detection of perforin and granzyme B in resting NK cells, cells were surface-stained with CD3 and CD56 at 4 °C for 20–30 min, then fixed with Medium A from the FIX & PERM Kit (Invitrogen, GAS004, USA) at room temperature for 10–20 min. Permeabilization and intracellular staining were performed in Medium B from the same kit using anti-perforin (BioLegend, clone dG9, USA) and anti-granzyme B (BioLegend, clone QA16A02, USA). Cells were then washed and resuspended in FACS buffer for flow cytometry acquisition. For NK cell functional assays, NK cells were co-incubated with K562 target cells at an effector-to-target ratio of 2:1 for 1 h in the presence of anti-CD107a (BioLegend, clone H4A3, USA). After incubation, cells were surface-stained with anti-CD3 and anti-CD56 at 4 °C for 20–30 min. Finally, cells were washed and resuspended in FACS buffer for acquisition. The percentage of CD107a-positive NK cells was background-corrected by subtracting basal degranulation levels from NK cells cultured without K562 target cells. The frequency of NK cells was defined as CD3^−^CD56^+^ population, and the expression levels of NKG2D, NKp44, NKp46, CD16, CD107a, perforin and granzyme B were analyzed within the gated CD3^−^CD56^+^ NK-cell subset.

### 2.5 Cytotoxicity assays

NK cell cytotoxicity was evaluated by measuring apoptosis of K562-GFP target cells (Procell, China) following co-culture. NK cells and K562-GFP cells were co-incubated at an effector-to-target (E:T) ratio of 5:1 for 1 h at 37 °C. K562-GFP cells cultured alone served as controls. After incubation, all cells were collected and stained with Annexin V-APC/PI (Keygen Biotech, China) at 4 °C for 10 min in the dark. Viable target cells were defined as Annexin V^−^/PI^−^. Cytotoxicity was calculated as:


Cytotoxicity (%)=(1–% viable K562 in coculture/% viable K562 in control)×100

### 2.6 Cell aggregation rate detection

Cell aggregation rate was measured using a Countstar automated cell analyzer after mixing cell suspensions with 0.4% trypan-blue solution at a 1:1 ratio. Sample identifiers were anonymized prior to measurement, and five independent microscopic fields were automatically captured by the software without manual intervention. Data acquisition and quantification were performed under blinded conditions. A cell aggregate was defined as a contiguous clump containing ≥3 viable cells. Trypan-blue-positive dead cells and dead-cell clumps were excluded from calculations. An 8–100 μm size threshold combined with morphological filters was applied to exclude feeder-cell debris, fibrin/fibrinogen fragments and irregular microparticulates. Air bubbles were filtered out based on circularity and hollow-morphology features identified by the software. The cell aggregation rate was calculated using the following formula:


Aggregation rate (%)=(viable cells in aggregates/total viable cells)×100%

### 2.7 Fibrinogen content analysis

Fibrinogen concentrations in human plasma samples were measured using a human fibrinogen ELISA kit (Abcam, UK) according to the manufacturer’s instructions. Plasma samples were diluted 1:1000 prior to analysis. All assays were performed three times. Fibrinogen concentrations were calculated from the standard curve using ELISACalc software.

### 2.8 Statistical analysis

Statistical analysis were performed using GraphPad Prism 8 (GraphPad Software, USA). Comparisons between two groups were conducted using unpaired Student’s t-tests. Statistical significance was set at P< 0.05, with significance levels denoted as *P< 0.05, **P< 0.01, and ***P< 0.001. All data are presented as mean ± standard deviation (SD) unless otherwise specified.

## 3 Results

### 3.1 NK cell products expanded with feeder cells develop non-dissociable aggregates

To establish a scalable production platform for NK cells, we engineered K562 cells to stably express 4-1BBL and membrane-bound IL-21 (mbIL-21) (**Supplementary Figures 1A, B**). Primary NK cells were expanded following the general procedure outlined in **Figure 1A**. Strikingly, macroscopic, non-dissociable aggregates were observed in the final umbilical cord blood (UCB)-derived NK cell products within 4 hours of storage at 2–8 °C (**Supplementary Figure 1C**), raising safety concerns for intravenous infusion, especially the microvascular occlusion or infusion-related adverse events.

**Figure 1:**
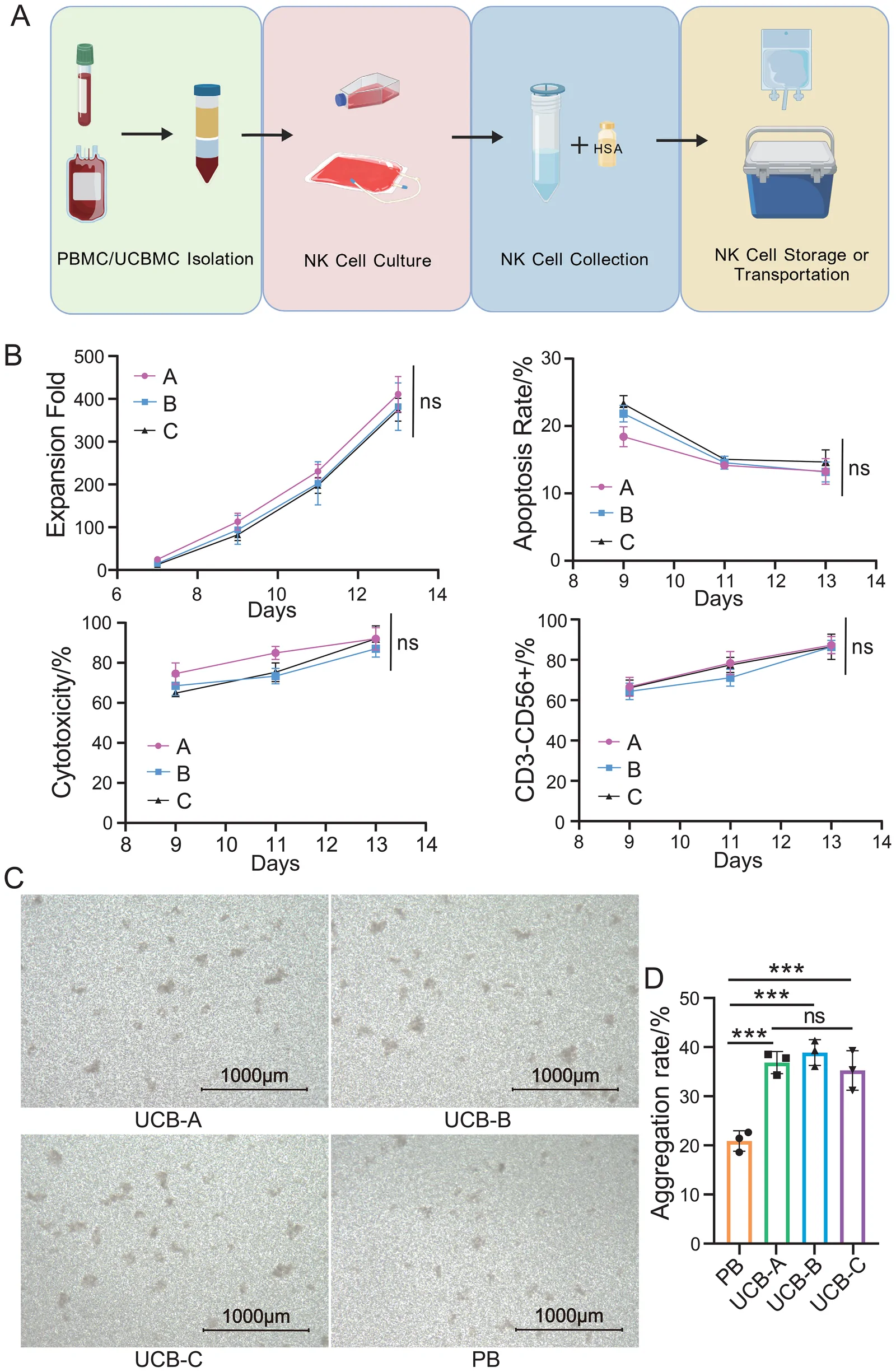
Feeder-expanded NK cells from multiple sources develop aggregates upon short-term storage. **(A)** Schematic overview of the NK cell expansion protocol using feeder cells. **(B)** Comparison of expansion fold, apoptosis rate, cell purity, and cytotoxic activity of umbilical cord blood-derived NK cells expanded with three different feeder cell systems. A: feeder cells developed by our group; B: feeder cells from Nuwacell Biotech; C: feeder cells from Yipin Biotech. **(C)** Representative microscopic images of cord blood-derived NK cell products expanded with three feeder cell systems and peripheral blood-derived NK cell products cultured with feeder cells developed by our group, after storage at 4 °C for 4 h. **(D)** Quantitative comparison of aggregation rates between peripheral blood- and umbilical cord blood-derived NK cell products after 4 hours of storage. Data are presented as mean ± SD from three independent human donors (n = 3), with each symbol corresponding to one donor. Representative microscopy images are shown from a single donor. ***P< 0.001; ns, not significant.

To determine whether this aggregation phenomenon was attributable to our self-developed feeder system, we compared it with two commercially available feeder cell lines under identical culture conditions. No significant differences were observed among the three groups in terms of expansion fold, purity, apoptosis rate, or cytotoxic activity (**Figure 1B**; **Supplementary Figures 1D–F**). Nevertheless, aggregates formation recurred in all NK cell products following formulation, as confirmed by microscopic examination (**Figure 1C**). We next assessed NK cells derived from peripheral blood (PB) expanded with feeder cells. Aggregates similarly developed after product formulation; notably, PB-derived cell aggregates were markedly smaller in size and lower in frequency than those observed in UCB-derived NK cells under equivalent storage conditions (**Figure 1D**). Quantitative analysis further revealed that the aggregation rate of UCB-derived NK cells was substantially higher than that of PB-derived NK cells (**Figure 1E**).

Collectively, these data indicate that neither the source of primary NK nor the feeder cells is the key determinant of cell aggregation.

### 3.2 Optimization of collection and formulation procedures is insufficient to prevent aggregate formation

Given that little irreversible aggregates were observed during NK cell culture process, we hypothesized that aggregate formation might be triggered during the cell collection procedure. To test this, we systematically modulated several key harvesting parameters. Firstly, centrifugal force was reduced from 500×g to 300×g. This adjustment led to a modest reduction in cell aggregation burden, although substantial aggregates remained. Conversely, increasing the centrifugal force to 800×g resulted in a marked increase in the aggregation rate, consistent with our expectations (**Figures 2A, B**). As we know, cell aggregates may continue to enlarge once formed. We next attempted to remove pre-formed tiny cell aggregates through a finer cell strainer. While the use of a smaller-pore strainer moderately reduced the size and quantity of aggregates, it failed to eliminate the problem completely (**Figures 2C, D**). We then examined the effect of cell concentration in the final product bag: NK cells were formulated at densities of 2×10^7^/mL, 3×10^7^/mL, or 4×10^7^/mL. Both aggregation rate and size were indeed increased progressively with higher cell concentrations (**Figures 2E, F**). In parallel, we evaluated the impact of human serum albumin (HSA) supplementation, which is a commonly used excipient for cell protection (19). NK products without HSA exhibited an huge aggregation rate of approximately 65% at 4 h, while increasing the HSA proportion did not reduce the aggregation rate obviously (**Figures 2G, H**). Finally, we integrated all the optimized parameters—centrifugal force of 300×g, 40-μm cell strainer, cell density of 2×10^7^/mL, and 1.5% HSA supplementation—to collect and formulate NK cells. Although this approach significantly reduced the initial aggregation at 4 h post-formulation compared with previous NK cell products, substantial aggregates re-emerged after 12 and 24 hours (**Figures 2I, J**). Moreover, the use of lower centrifugal force and finer cell strainers was associated with increased cell loss, compromising final product yield.

**Figure 2:**
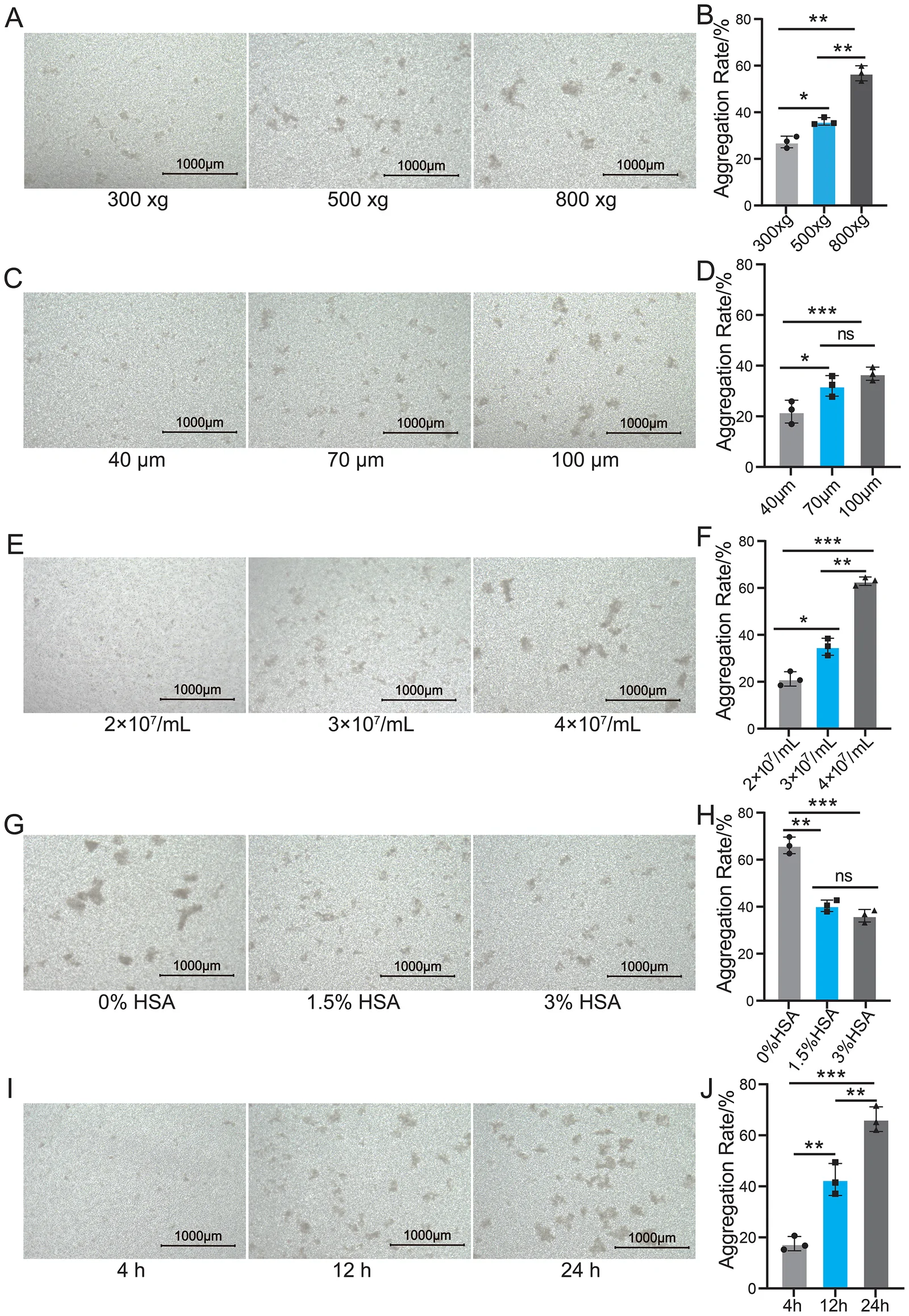
Optimization of collection parameters is insufficient to prevent aggregate formation. Representative microscopic images **(A)** and aggregation rates **(B)** in NK cell products harvested with different centrifugal forces (300×g, 500×g, 800×g) after 4 hours of storage. Representative microscopic images **(C)** and aggregation rates **(D)** of NK cell products filtered through cell strainers of varying pore sizes (40 μm, 70 μm, 100 μm) after 4 hours of storage. Representative microscopic images **(E)** and aggregation rates **(F)** of NK cell products formulated at different cell densities (2 × 10^7^/mL, 3 × 10^7^/mL, 4 × 10^7^/mL) after 4 hours of storage. Representative microscopic images **(G)** and aggregation rates **(H)** of NK cell products supplemented with different concentrations of HSA (0%, 1.5%, 3%) after 4 hours of storage. Representative microscopic images **(I)** and aggregation rates **(J)** of NK cell products harvested under optimized conditions (300×g, 40 μm, 2 × 10^7^ cells/mL, 1.5% HSA) after 4, 12, and 24 hours of storage. Data are presented as mean ± SD from three independent human donors (n = 3). Representative microscopy images are from a single donor. *P< 0.05, **P< 0.01, ***P< 0.001; ns, not significant.

Collectively, these findings demonstrate that while certain collection and formulation parameters influence the extent of NK cell aggregation, the phenomenon cannot be fully resolved through procedure optimization alone. The persistence of aggregates under clinically relevant storage conditions, underscores the need for alternative strategies to ensure product stability and safety.

### 3.3 Heparin fails to prevent NK cell aggregate formation

Given the persistent aggregation observed in our NK cell products after collection optimization, we hypothesized that coagulation factors in the culture system might contribute to this phenomenon. Heparin is a commonly used anticoagulant in stem cell expansion to inhibit aggregate formation (20–22). We therefore investigated whether heparin supplementation during NK cell expansion could mitigate the aggregation problem.

Heparin was added to NK cell culture system at concentrations of 0, 1, 5, or 10 IU/mL. Contrary to expectation, high-dose heparin (5 and 10 IU/mL) induced markedly macroscopic and non-dissociable aggregate formation during culture period. These aggregates were distinct from the physiological, transient NK cell clustering associated with NK cell activation (**Figure 3A**). Consistent with this observation, NK cell proliferation was significantly impaired at higher heparin concentrations (**Figure 3B**). Moreover, elevated heparin levels reduced the purity of the expanded NK cell populations (**Figures 3C, D**), although no significant differences were detected in cytotoxic activity or apoptosis (**Supplementary Figures 2A, B**). Following product formulation, the aggregation rate was modestly reduced in the 1 IU/mL heparin group compared to untreated control. In contrast, the 5 IU/mL and 10 IU/mL heparin groups exhibited substantially higher aggregation rates (**Figures 3E, F**).

**Figure 3:**
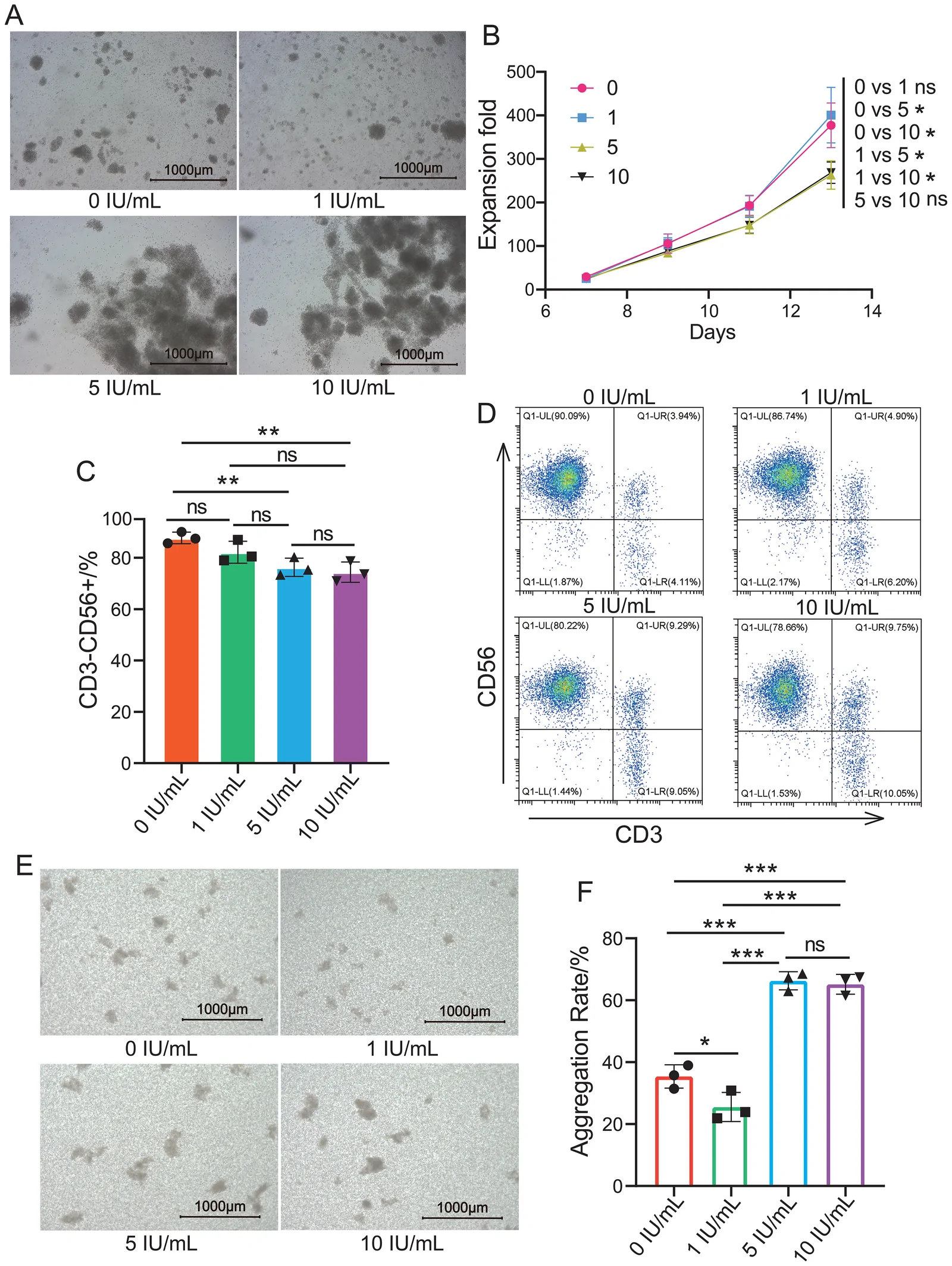
Heparin supplementation fails to prevent NK cell aggregation. **(A)** Representative microscopic images of NK cell supplemented with 0, 1, 5, or 10 IU/mL heparin during expansion. Expansion fold **(B)** and purity **(C, D)** of NK cells expanded with 0, 1, 5, or 10 IU/mL heparin. Representative microscopic images **(E)** and quantification of aggregation rates **(F)** in NK cell products expanded with 0, 1, 5, or 10 IU/mL heparin after 4 hours of storage post-formulation. Data are presented as mean ± SD from three independent human donors (n = 3). Representative flow-cytometry plots and microscopy images are from a single donor. *P< 0.05, **P< 0.01, ***P< 0.001; ns, not significant.

These findings suggest that while heparin possesses some capacity to mitigate aggregation, a concentration of 1 IU/mL proved insufficient, whereas higher concentrations adversely disrupted NK cell expansion and purity. Accordingly, heparin supplementation is not a suitable strategy for preventing aggregate formation in feeder-expanded NK cell cultures.

### 3.4 Feeder cell drives aggregate formation in NK cell products

Since optimization of the collection procedure only alleviated but did not resolve the aggregation issue, we reasoned that initiating factors for aggregate formation might already be present during the culture process. Consistent with this, the addition of anticoagulant, heparin, in the culture medium could moderately decrease the aggregation rate, though excessive heparin resulted in impaired cell activity. Apoptotic cells are known to release large quantities of DNA and histones, which can promote cell aggregation (23, 24). Notably, feeder cells undergo rapid apoptosis upon coculture with NK cells (25), generating abundant cellular debris rich in these pro-aggregatory molecules.

To investigate whether feeder cell debris contributes to aggregate formation, we first adjusted the feeder cell amount added at day 7 (the second feeder addition). Reducing the number of feeder cells did not exert a significant influence on the expansion fold, purity, or cytotoxicity of NK cells (**Figures 4A–C**).

**Figure 4:**
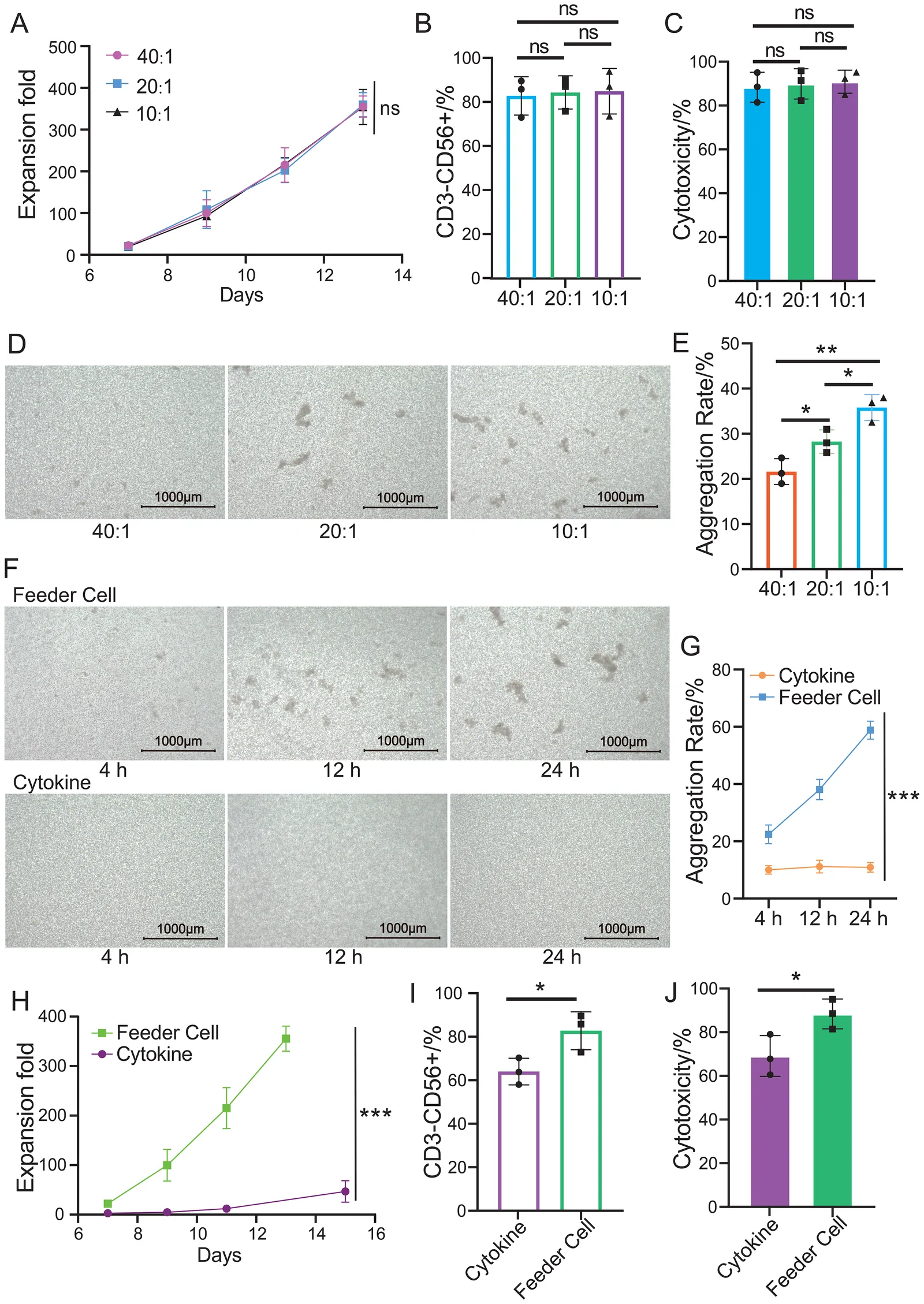
Feeder cells contribute to aggregate formation in NK cell products. Expansion fold **(A)**, Purity **(B)** and cytotoxic activity **(C)** of NK cells expanded with varying feeder cell ratios. Representative microscopic images **(D)** and quantification of aggregation rates **(E)** in NK cell products expanded with varying feeder cell ratios after 4 hours of storage. Representative microscopic images **(F)** and quantification of aggregation rates **(G)** in feeder-expanded versus cytokine-expanded NK cell products after 4, 12, and 24 hours of storage. Comparison of expansion fold **(H)**, purity **(I)** and cytotoxic activity **(J)** between feeder-expanded and cytokine-expanded NK cells. Data are presented as mean ± SD from three independent human donors (n = 3). Representative microscopy images are from a single donor. *P< 0.05, **P< 0.01, ***P< 0.001; ns, not significant.

Strikingly, aggregate incidence in the final NK cell product decreased markedly in a dose-dependent manner as feeder cell numbers were lowered (**Figures 4D, E**). However, aggregate formation was not entirely eliminated under these conditions, suggesting that residual feeder-derived factors or another mechanisms remain operative. Encouraged by the partial benefit of feeder cell reduction, we next employed a feeder-free culture system. NK cells expanded with cytokines alone were virtually free of aggregates, and no aggregation developed even after prolonged storage (**Figures 4F, G**). Crucially, however, it cannot be ignored that NK cells expanded with feeder system conferred a substantial advantage in terms of NK cell expansion yield, purity and cytotoxicity (**Figures 4H–J**).

Collectively, these observations implicate feeder cells as a key triggering factor in aggregate formation. Nevertheless, considering the superior expansion efficacy of the feeder-based system, merely abstaining from using feeder cells is not a viable approach for establishing a large-scale NK cell production platform. Therefore, strategies aimed at alleviating aggregation should be explored within the context of feeder-dependent NK cell expansion.

### 3.5 Fibrinogen depletion eliminates aggregates in feeder-expanded NK cell products

Fibrinogen, a component present in human platelet lysate (hPL) and plasma commonly used in cell culture, has been reported to promote cell aggregation (26, 27). Given its structural capacity to bind integrins and DNA/histone complexes, we hypothesized that fibrinogen/fibrin acts synergistically with feeder-derived apoptotic debris to promote aggregation in our NK cell cultures.

To investigate this, we quantified fibrinogen levels in plasma subjected to different treatments using ELISA. Three conditions were evaluated: untreated plasma (u-P), frozen-thawed plama (ft-P), and commercial fibrinogen-depleted human platelet lysates (fd-hPL; fibrinogen concentration<20 μg/mL). Ft-P was the plasma applied in above research, since repeated freeze-thaw cycles are a general method to reduce fibrinogen content of plasma in research scale (26). As expected, fibrinogen levels were significantly lower in fd-hPL compared to both u-P and ft-P (**Figure 5A**). We next cultured NK cells using these three supplements. Notably, NK cell products generated using fd-hPL were completely free of visible aggregates. In contrast, aggregates exhibited significantly higher abundance in the u-P group compared to the ft-P group. (**Figure 5B**), indicating that aggregates formation occurs in a fibrinogen concentration dependent manner. Regarding expansion efficacy, no significant differences were observed among these groups in terms of NK cell expansion fold, purity, or cytotoxic activity (**Figures 5C–E**). Likewise, no significant differences were observed in the expression levels of surface activating receptors (CD16, NKp44, NKp46, NKG2D), degranulation marker (CD107a), or intracellular cytotoxic effector proteins (granzyme B, perforin) among NK cells cultured with the three supplements. (**Figure 5H**; **Supplementary Figure 3**). Importantly, NK cells expanded with fd-hPL remained aggregate-free throughout storage, with no visible and dissociable aggregates detected at 4, 12, or 24 hours post-formulation (**Figures 5F, G**).

**Figure 5:**
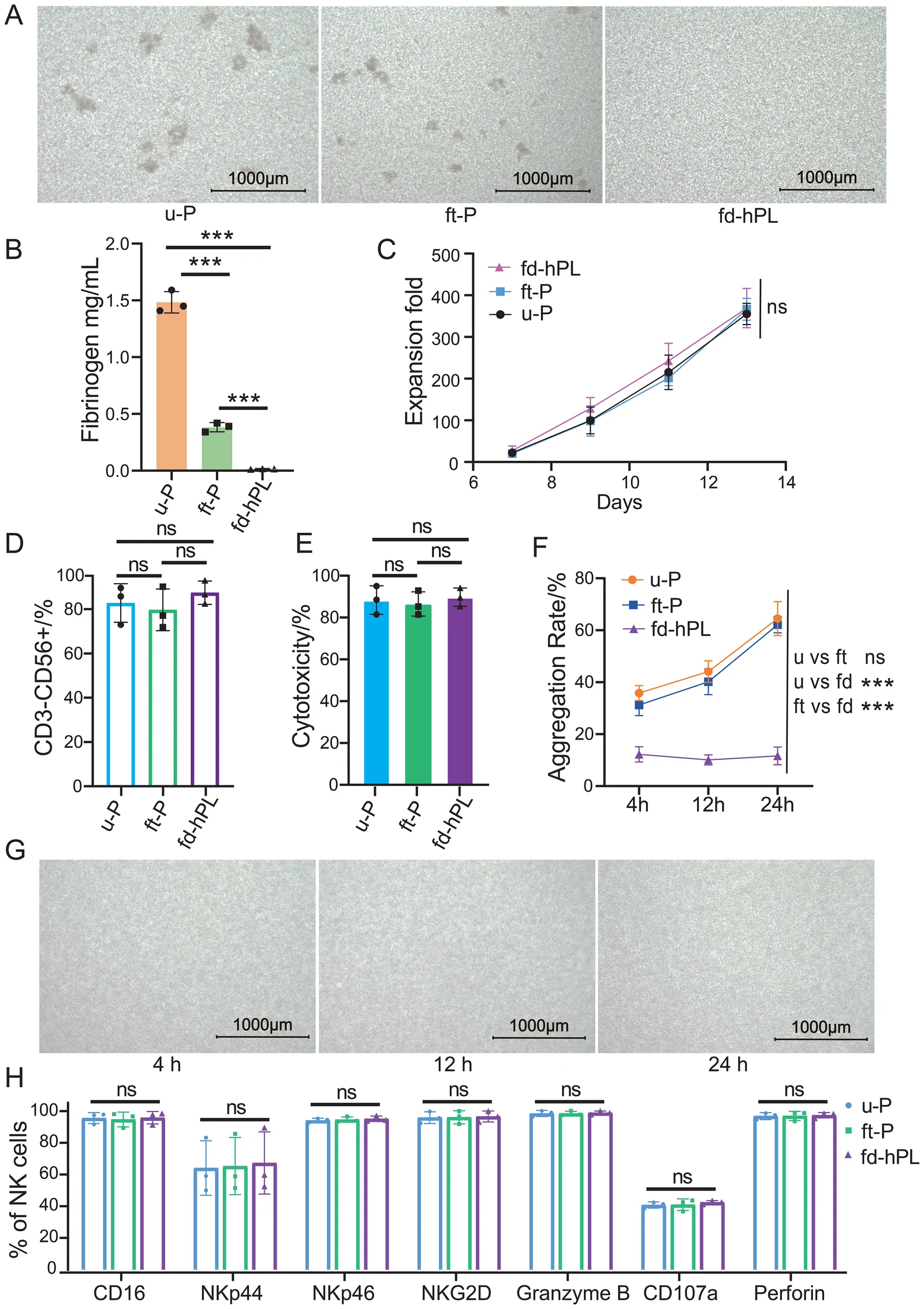
Fibrinogen depletion eliminates aggregate formation in feeder-expanded NK cell products. **(A)** Representative microscopic images of NK cell products generated using u-P, ft-P, or fd-hPL after 4 hours of storage. **(B)** Quantification of fibrinogen concentrations in three plasma preparations: untreated plasma (u-P), frozen-thawed plasma (ft-P), and fibrinogen-depleted human platelet lysate (fd-hPL). Comparison of NK cell expansion fold **(C)**, purity **(D)**, and cytotoxic activity **(E)** in NK cells cultured with u-P, ft-P, or fd-hPL. **(F)** Time-course quantification of aggregation rates in NK cell products generated using u-P, ft-P, or fd-hPL after 4, 12, and 24 hours of storage. **(G)** Representative microscopic images of NK cell products generated using fd-hPL after 4, 12, and 24 hours of storage. **(H)** Comparison of the expression levels of surface activating receptors (CD16, NKp44, NKp46, NKG2D), degranulation marker (CD107a), and intracellular cytotoxic effector proteins (granzyme B, perforin) in NK cells cultured with u-P, ft-P, and fd-hPL. Data are presented as mean ± SD from three independent human donors (n = 3). Representative microscopy images are from a single donor. ***P< 0.001; ns, not significant.

Taken together, these results demonstrate that fibrinogen is the key determinant of aggregate formation in feeder-expanded NK cell products, and that its depletion effectively prevents aggregation without compromising NK cell yield or function.

## 4 Discussion

In recent years, the growing clinical demand for NK cell-based therapies has highlighted the urgent need for standardized and reproducible manufacturing platforms. Among existing approaches, feeder cell-based expansion systems have demonstrated particular efficacy, enabling robust and large-scale production of NK cells with high purity and potent cytotoxic activity (4).

In this study, we first established a feeder-based expansion protocol for generating NK cells from both umbilical cord blood and peripheral blood. However, macroscopic cell aggregation was consistently observed in the final NK cell products after storage. Notably, these aggregates increased in both size and number with extended storage time. Distinct from the transient, reversible NK-cell clusters formed during activation or feeder-cell interactions, these aggregates could not be dispersed by gentle shaking or pipetting. Such aggregation poses a significant concern for clinical safety: infusion of products containing aggregates at higher cell doses may increase the risk of adverse events, including dyspnea, chills, hypoxia, and potentially thromboembolic complications (28, 29). While such symptoms have traditionally been attributed to immune activation or infusion-related reactions, our findings raise the possibility that these unnoticed cell aggregation contribute to these clinical manifestations.

Cell aggregates can emerge at multiple stages of the manufacturing process, including during NK cell expansion and harvest. During the expansion stage, however, no abnormal aggregation was observed, leading us to hypothesize that aggregate formation primarily occurs during or after cell collection. Accordingly, we focused on several key parameters associated with the harvesting procedure: centrifugal force, cell density, pore size of cell strainers, and HSA concentration. Accordingly, we focused on several key parameters associated with the harvesting procedure: centrifugal force, cell density, pore size of cell strainers, and HSA concentration. Consistent with prior studies showing that elevated centrifugal forces compromise membrane integrity and reduce viability (30, 31), we observed a force-dependent increase in aggregation incidence. Filtration through 40-μm strainers effectively removed pre-existing aggregates, and reducing the final cell concentration also markedly decreased aggregation frequency. In addition, HSA is well-established as a stabilizing agent that mitigates shear-induced membrane perturbation (32); accordingly, omission of HSA from the formulation buffer resulted in extensive, irreversible aggregation. However, increasing HSA concentration beyond 1.5% (w/v) conferred no additional benefit. We next combined these optimized parameters (300×g centrifugation, 40-μm filtration, 2 × 10^7^ cells/mL, and 1.5% HSA) and observed a transient improvement in aggregation status. This partial improvement is mechanistically plausible: mechanical shear stress during centrifugation may induce cell lysis, releasing intracellular damage-associated molecular patterns (DAMPs) such as histones and DNA that promote intercellular cross-linking and aggregation (33). Filtration removes larger pre-formed aggregates, thereby limiting further growth, while reduced cell density minimizes cell–cell contact and subsequent cross-linking. Nevertheless, aggregation gradually reappeared with extended storage time. These findings indicate that while procedural modifications can mitigate aggregation to some extent, they do not address the underlying cause and may compromise final product yield.

We next examined components of the culture system. Soluble proteins present in culture supplements can gradually interact with cellular debris released from dying cells to form insoluble aggregates. Although heparin is routinely added to stem cell cultures to inhibit flocculation and aggregation (20–22), its utility in NK cell expansion systems remains unexplored. We therefore evaluated the effect heparin supplementation throughout NK cell expansion. Strikingly, high heparin concentrations (5 and 10 IU/mL) paradoxically induced substantial aggregation even during the culture period, accompanied by significantly impaired NK cell proliferation and reduced cell purity. Although previous studies have reported cytotoxic effects of heparin (34), we observed no significant differences in NK apoptosis or cytotoxicity, possibly because aggregated cells were removed during processing and thus excluded from analysis. Low-dose heparin (1 IU/mL) modestly reduced post-formulation aggregation but failed to prevent it entirely. These findings indicate that heparin supplementation is not a suitable strategy for preventing aggregate formation in feeder-expanded NK cell cultures.

Given that feeder cells undergo rapid and extensive apoptosis upon co-culture with NK cells, we hypothesized that apoptotic debris—rich in extracellular DNA, histones, and phosphatidylserine-exposing membranes—may serve as nucleation sites for aggregation (23, 24). Consistent with this, reduction of feeder cells led to a significant decrease in aggregate formation, although aggregates were not completely eliminated. In contrast, NK cells expanded using a cytokine-only feeder-free system showed no irreversible aggregation. Therefore, feeder cells may be the key factor that result in cell aggregation.

Fibrinogen, a component present in human platelet lysate and plasma used for cell culture, has been reported to promote aggregation (26, 27). We therefore posited that fibrinogen acts synergistically with feeder-derived debris to drive aggregate formation. Consistent with this hypothesis, NK cell products generated using fibrinogen-depleted human platelet lysates, a commercially available reagent, were completely free of visible aggregates, and no aggregation developed even after prolonged storage. In contrast, products prepared with untreated plasma or frozen-thawed plasma—which retains residual fibrinogen despite repeated freeze-thaw cycles—contained abundant aggregates. These findings demonstrate that fibrinogen is a key determinant of aggregate formation in feeder-expanded NK cell products. Of note, these fibrinogen-mediated aggregation risks are not confined to NK cell cultures but also apply to the expansion of other cells, including mesenchymal stromal cells (MSCs). Previous studies have demonstrated that fibrinogen-depleted hPL can similarly reduce aggregate formation during MSC manufacturing (26, 27).

Despite these advances, several limitations warrant futher acknowledgment. First, the precise composition of aggregates remains uncharacterized; transmission electron microscopy will be required to confirm the presence of specific cellular debris and fibrinogen-derived components within the aggregates. Secondly, the reason why abnormal aggregates were only observed during the harvest and storage periods rather than the expansion stage remains unclear. It is hypothesized that this phenomenon can be attributed to the large volume of the culture medium and low cell density, which restricts intercellular contact and the efficiency of cross-linking required for aggregate formation. Thirdly, the underlying mechanism of marked difference in aggregation propensity between PB- and UCB-derived NK cells—despite identical manufacturing parameters—remains to be elucidated. This disparity is highly likely attributable to differential anticoagulant exposure: heparin (used for PB collection) potently inhibits thrombin-mediated fibrin polymerization, whereas sodium citrate (used for UCB collection) chelates calcium and only partially suppresses coagulation cascade activation. And prior evidence also indicates that sodium citrate may impair mononuclear cell viability, potentially exacerbating apoptosis and promoting aggregation (35). In addition, NK cell lines such as NK-92 and NK101 can serve as alternative NK cell sources for clinical therapy. These cell lines can be readily expanded ex vivo and do not require feeder cells (36, 37). Thus, the present study focuses on aggregation-related challenges in feeder-based primary NK cell manufacturing.

Collectively, these results demonstrate that aggregate formation in NK cell products arises from the combined effects of apoptotic debris derived from feeder cells and fibrinogen present in the plasma (summarized in **Figure 6A**) (38). Consequently, either eliminating feeder cells or depleting fibrinogen resolves aggregation—but only the latter preserves the superior expansion capacity, cell purity and cytotoxic potency conferred by feeder support. Thus, substitution of plasma with fibrinogen-depleted alternatives represents a pragmatic, GMP-compatible optimization. Thus, an overview of the optimized and validated expansion protocol is provided in **Figure 6B**. Briefly, NK cells were activated and expanded with feeder cells, supplemented with fibrinogen-depleted hPL, harvested via centrifugation at 500×g for 10 min, filtered through a 100-μm cell strainer, and formulated with 1.5% HSA at 3 × 10^7^ cells/mL.

**Figure 6:**
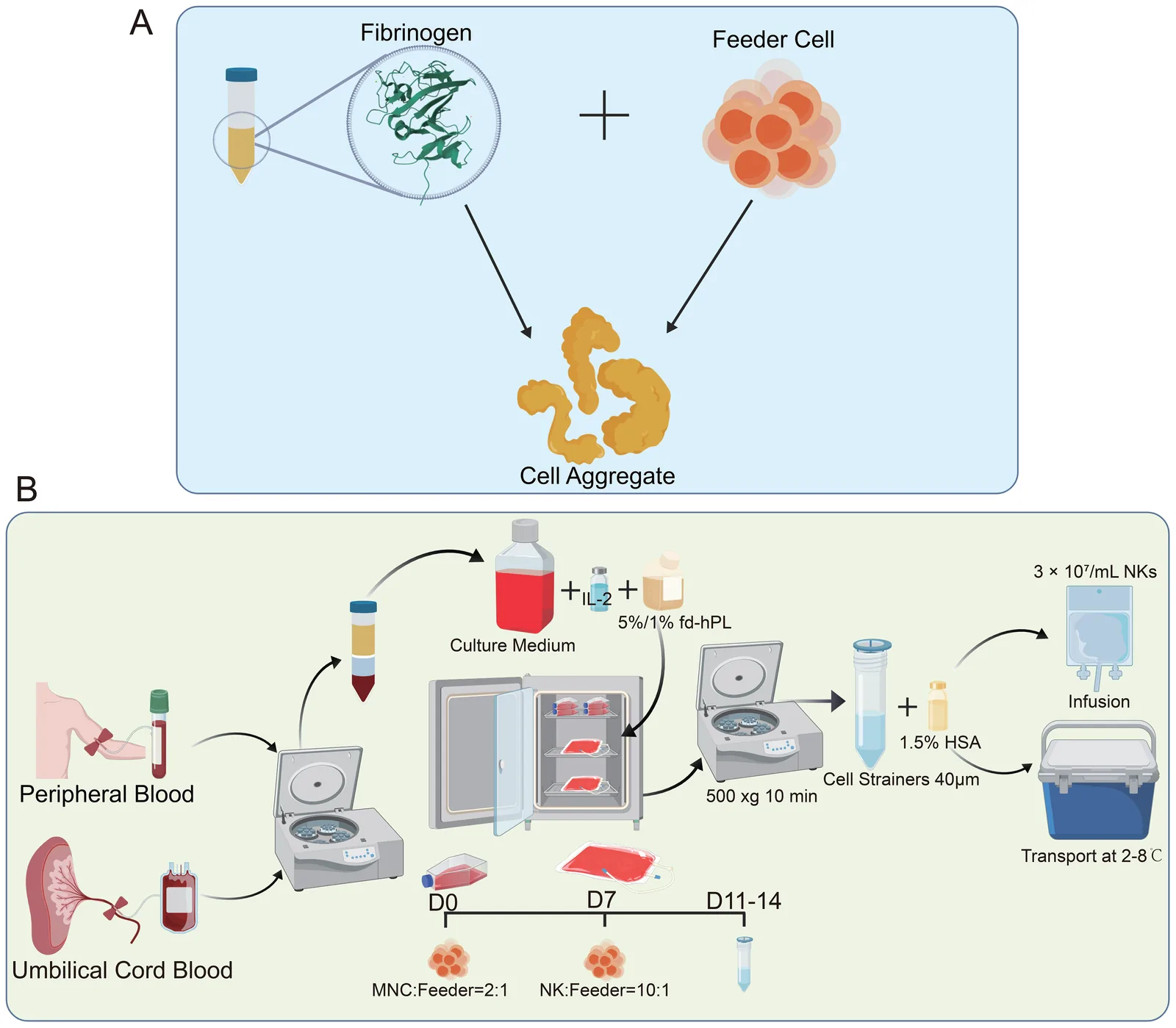
Mechanism of aggregate formation and optimized protocol for NK cell manufacturing. **(A)** Schematic summary illustrating the synergistic contribution of feeder cell and plasma fibrinogen to aggregate formation in NK cell products. **(B)** Graphical overview of the optimized NK cell expansion and formulation protocol. The diagram was created using BioGDP.com.

## 5 Conclusions

This study identifies fibrinogen as a critical and previously unrecognized factor contributing to aggregate formation in feeder-expanded NK cell products. By replacing standard plasma or platelet lysate with fibrinogen-depleted supplements, the aggregation problem can be effectively resolved without compromising NK cell yield, purity, or activity. This simple, scalable, and immediately translatable intervention enhances product safety, stability, and infusion reliability—thereby advancing the clinical feasibility and regulatory readiness of off-the-shelf NK cell therapies.

## Data Availability

The original contributions presented in the study are included in the article/**Supplementary Material**. Further inquiries can be directed to the corresponding authors.
