# Supplementary material for: Fibrinogen depletion prevents non-dissociable cell aggregates of feeder-derived NK cell products
Source: Front Immunol. 2026 Sep 16;17:1887148. doi: 10.3389/fimmu.2026.1887148 (PMC13623860; doi:10.3389/fimmu.2026.1887148)
Supplement: Supplementary file 1 [file Supplementaryfile1.docx]

**Supplementary Figures**

Supplementary Figure 1. Characterization of Feeder Cells and NK Cell Product Aggregation Kinetics. (A) Schematic diagram illustrating the construction of feeder cells. (B) Flow cytometry analysis of surface expression of 4-1BBL and mbIL-21 on engineered K562 feeder cells. (C) Representative microscopic images of NK cell products after storage at 4°C for 4, 12, and 24 hours. (D–F) Representative flow cytometry plots comparing (D) purity, (E) apoptosis rate (Annexin V/PI staining), and (F) cytotoxic activity against K562-GFP target cells among NK cells expanded with three different feeder cell sources. A: feeder cells developed by our group; B: feeder cells from Nuwacell Biotech; C: feeder cells from Yipin Biotech.

Supplementary Figure 2. Effect of Heparin Supplementation on NK Cell Apoptosis and Cytotoxicity. (A) Representative flow cytometry plots (Annexin V/PI staining) showing apoptosis of NK cells expanded with 0, 1, 5, or 10 IU/mL heparin. (B) Representative flow cytometry plots assessing NK cell cytotoxic activity against K562-GFP target cells following expansion with 0, 1, 5, or 10 IU/mL heparin. Target cell apoptosis was measured by Annexin V-APC/PI staining.

Supplementary Figure 3. Representative flow cytometry plots showing the expression profiles of surface activating receptors and functional markers on NK cells cultured with fd-hPL.
